# Avian species diversity and transmission of West Nile virus in Atlanta, Georgia

**DOI:** 10.1186/s13071-017-1999-6

**Published:** 2017-02-03

**Authors:** Rebecca S. Levine, David L. Hedeen, Meghan W. Hedeen, Gabriel L. Hamer, Daniel G. Mead, Uriel D. Kitron

**Affiliations:** 1 Department of Environmental Sciences, Emory University, 400 Dowman Drive, Math and Science Center 5th Floor, Suite E510, Atlanta, GA 30322 USA; 2 Georgia Department of Transportation, Office of Environmental Services, One Georgia Center, 600 West Peachtree Street NW, Atlanta, GA 30308 USA; 3 Department of Entomology, Texas A&M University College of Agriculture and Life Sciences, TAMU 2475, College Station, TX 77843 USA; 4 University of Georgia College of Veterinary Medicine, Southeastern Cooperative Wildlife Disease Study, 589 D.W. Brooks Drive, Athens, GA 30602 USA

**Keywords:** Dilution effect, Amplification, Host competence, Community composition, West Nile virus, Northern cardinal

## Abstract

**Background:**

The dilution effect is the reduction in vector-borne pathogen transmission associated with the presence of diverse potential host species, some of which are incompetent. It is popularized as the notion that increased biodiversity leads to decreased rates of disease. West Nile virus (WNV) is an endemic mosquito-borne virus in the United States that is maintained in a zoonotic cycle involving various avian host species. In Atlanta, Georgia, substantial WNV presence in the vector and host species has not translated into a high number of human cases.

**Methods:**

To determine whether a dilution effect was contributing to this reduced transmission, we characterized the host species community composition and performed WNV surveillance of hosts and vectors in urban Atlanta between 2010 and 2011. We tested the relationship between host diversity and both host seroprevalence and vector infection rates using a negative binomial generalized linear mixed model.

**Results:**

Regardless of how we measured host diversity or whether we considered host seroprevalence and vector infection rates as predictor variables or outcome variables, we did not detect a dilution effect. Rather, we detected an amplification effect, in which increased host diversity resulted in increased seroprevalence or infection rates; this is the first empirical evidence for this effect in a mosquito-borne system.

**Conclusions:**

We suggest that this effect may be driven by an over-abundance of moderately- to poorly-competent host species, such as northern cardinals and members of the Mimid family, which cause optimal hosts to become rarer and present primarily in species-rich areas. Our results support the notion that dilution or amplification effects depend more on the identities of the species comprising the host community than on the absolute diversity of hosts.

**Electronic supplementary material:**

The online version of this article (doi:10.1186/s13071-017-1999-6) contains supplementary material, which is available to authorized users.

## Background

Community ecology focuses on the interaction, distribution, abundance, and demography of coexisting populations of diverse species. Modern community ecology examines patterns and processes that occur between two or more species inhabiting the same geographical area. For zoonotic and vector-borne pathogens whose transmission cycles often involve multiple hosts, interactions between vectors and hosts involve complex dynamics that determine disease risk.

For over 100 years, the notion that increased species diversity is linked with reduced disease transmission has been recognized. This phenomenon was initially observed in the protective effect that the presence of domestic animals had in reducing human mosquito-biting rates and malaria transmission (reviewed by [1]). That the presence of additional species could reduce vector-borne disease transmission to humans by providing blood-meals to hematophagous arthropods from dead-end hosts was recognized and put into practice long before the World Health Organization (WHO) defined this practice in 1982 as “zooprophylaxis” [1–3]. Although zooprophylaxis has been employed to reduce pathogen transmission to humans, the practice remains controversial because increasing the presence of domestic animals around human habitations can also increase the abundance of various blood-feeding vectors through the provision of additional blood resources [1–4].

While vector biologists and entomologists were examining the effects of zooprophylaxis and its contribution to human vector-borne disease reduction, parasitologists were examining a similar effect for diseases associated with free-living parasites. After discovering that the presence of various non-host snail species reduced the frequency of *Schistosmoa mansoni* infection among host snails, parasitologists proposed the “decoy effect” [5]. Repeated testing has shown that free-living parasites have a decreased ability to locate and/or infect their target hosts in the presence of additional non-host species [6–11]. Like zooprophylaxis, the outcome of the decoy effect in terms of pathogen transmission has not been consistent, with certain free-living parasites exhibiting no reduction [12] or increased amounts [7, 13] of infection-interference through the presence of additional hosts.

Though the link between increased species diversity and potential disease reduction quietly percolated in the fields of parasitology and medical entomology for well over half a century, it has only been brought to the forefront of disease and community ecology research in the past decade under the designation of the “dilution effect”. Beginning with a series of theoretical models coupled with empirical studies on the infection rates of Lyme disease in the Northeastern United States in the late 1990′s [14–16], the dilution effect was officially defined in 2000 by Ostfeld and Keesing as the reduction in vector-borne pathogen transmission that occurs through the presence of a diverse set of potential host species, some of which are relatively or completely incompetent as hosts [17, 18]. In order for the dilution effect to apply to a system, the following conditions must necessarily be met: (i) the vector is a generalist and feeds on a variety of host species, (ii) the vector becomes infected with the pathogen from its hosts, (iii) the different host species vary in their abilities to infect the vector (reservoir competence), and (iv) the hosts that are the most competent reservoirs tend to be dominant in the community [17]. Though not a universal phenomenon, there is evidence from natural, experimental, and theoretical studies on multiple systems of vector-borne pathogens for the existence of dilution of infectious disease in species rich communities [19–23], more research is needed to better understand both patterns and processes that result in dilution effects or their absence [24–26].

Since its introduction to the continental United States in 1999, the vector-borne and zoonotic West Nile virus (WNV) has become enzootic and endemic, resulting in an estimated 780,000 illnesses and 1,900 deaths [27, 28] along with population-level impacts on several bird species [29]. WNV transmission occurs between vectors (*Culex* mosquitoes) and competent hosts (passerine birds), with mammals representing dead-end hosts for the virus. Four empirical studies testing whether the dilution effect exists within the WNV system have been conducted to date. Two of these studies were conducted on the relatively coarse-scale of a regional and national level and both found evidence for the existence of a dilution effect in the WNV system [30, 31]. The two other studies were conducted on the relatively fine-scale of the county and metropolitan area and one found evidence for the dilution effect in the WNV system [21] while the other did not [32].

Because these study findings demonstrated no consistent pattern of a dilution effect in the WNV system, especially at fine scales, we sought to test the dilution effect for WNV at a fine-scale in a previously untested location in the USA with low rates of human disease. In Georgia, substantial WNV presence in the vector and host species has not translated into many human cases [27, 33]. In Atlanta, Georgia’s major urban center, yearly routine mosquito surveillance has consistently demonstrated active WNV infection in *Culex* mosquitoes [34] and both passive dead bird surveillance and active live bird surveillance have also indicated yearly WNV infection among avian hosts in Atlanta at levels consistent with rates found in other urban centers such as Chicago [34–38]. However, a total of only 330 human cases have been reported in Georgia since 2001 [33].

The goal of this study was to test for a dilution effect among the avian host and mosquito vector species in urban Atlanta, GA, USA, to determine whether this type of effect was contributing to reduced WNV spillover transmission to humans. To this end, we conducted comprehensive multi-season, multi-habitat, characterization of the avian species community as well as longitudinal WNV surveillance of avian hosts and mosquito vectors in urban Atlanta.

## Methods

### Study area

**Fig. 1:**
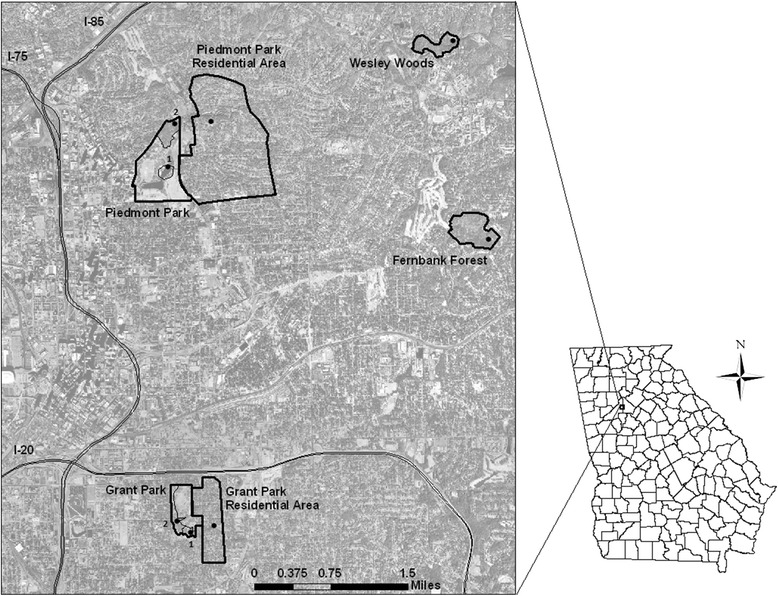
Map of the eight study sites in urban Atlanta, GA, USA, 2010–2011. Grant and Piedmont Parks each included two sampling zones, outlined within the park borders: (1) a water feature and surrounding structures; (2) a wooded area and associated walking paths. Survey points within each site are also shown. Copyright: Licensed to Mary Ann Liebert, Inc., New Rochelle, NY and reprinted in part with permission. Citation: Levine et al (2013) Limited spillover to humans from West Nile Virus viremic birds in Atlanta, Georgia: Fig. 1, *Vector-Borne and Zoonotic Diseases* 2013, 13: 11, pp. 812–817

Between early May and early November of 2010–2011, we trapped mosquitoes and wild passerine and near-passerine birds in 4 urban microhabitats of Atlanta, GA, USA: mixed-use parks, divided into wooded and water sections; residential areas; and old-growth forest patches (Fig. 1). The park and residential sites were treated as matched blocks, with residential sampling conducted in the neighborhoods directly east of the parks. Parks were divided into two zones: Park-Water contained an artificial water feature (pond or lake) surrounded by public restrooms and other built facilities (public swimming pool, tennis courts, gazebos, or large parking lots); Park-Woods comprised a wooded area with paved walking paths that experienced far less human use. Sampling frequency is described in Additional file 1.

### Field sampling

Wild birds were captured using nylon mesh mist nets [39]. Briefly, after extraction, captured birds were identified to species [40], measured, aged when possible to “hatch-year” or “after hatch-year” [41], sexed when possible [41], banded [42], blood-sampled (by jugular venipuncture), and released. After blood collection, samples were maintained on ice and centrifuged. Serum was then collected and frozen at -80 °C until further processing. Certain individuals were captured more than once. When possible, measurements and blood were obtained during each recapture to examine WNV seroprevalence status over time; however, to avoid pseudoreplication, status from only the first capture event was used in subsequent analyses [32].

To measure avian diversity, 10 min unlimited-radius point counts [43] were conducted at each site by expert observers [39]. A single point in each site was counted once per month (June-October in 2010 and May-October in 2011, Fig. 1). Although observers recorded all detected individuals, birds observed only flying over survey sites were not included in further analysis as they could not be determined to be living and breeding in that habitat.

Mosquitoes were captured using CDC gravid and light traps [39]. Gravid traps were baited with a hay and dog-food infusion and light traps were baited with CO_2_ in the form of dry ice [44, 45]. A trap session at each site consisted of 3 gravid traps and 1 light trap deployed throughout the site at or shortly before dusk and collected the following morning. Following collection, mosquitoes were identified to sex and species [B. Harrision, Keys to the mosquitoes of the Mid-Atlantic Region, unpublished] and inspected for presence of blood-meals. Because *Culex quinquefasciatus* and *C. restuans* co-occur in the area and cannot reliably be separated based on morphological characteristics alone, [T. McKinnish, B. Harrison, K. Caillouet, M. Hutchinson, B. Byrd, 2013, Validity of morphological characters used to distinguish *Culex restuans* and *Culex pipiens*, unpublished] we only identified *Culex* mosquitoes to the genus level. Up to 25 non-blood-fed females of the same species from the same trap (site, date) were pooled together in virus isolation media and frozen at -80 °C until further processing.

### Laboratory analyses

Avian sera were tested for antibodies to WNV using an epitope-blocked enzyme-linked immunosorbent assay (b-ELISA), as previously described [38, 39]. Briefly, this inhibition assay consisted of a sandwich containing a monoclonal capture antibody, a WNV recombinant antigen, a labeled monoclonal antibody, and avian serum. Following multiple incubations and washes, reduction in optical density of each sample was determined and percent inhibition calculated. All avian sera were initially screened at a dilution of 1:20. Samples testing positive in the initial screen were serially diluted (up to 1:640) and re-screened to confirm results and determine endpoint titers.

Mosquitoes were screened for circulating virus through virus isolation in cell culture [39]. Mosquito pools were homogenized and the supernatant fluid was inoculated onto Vero E6 cell cultures. Cells were visualized daily for two weeks and inspected for evidence of cytopathic effects (CPE). If CPE were noted, cultures were tested for WNV antigen using the Vector Test™ WNV Antigen Assay [46]. Viral RNA was extracted from Vector Test positive samples and identification was confirmed by reverse transcription PCR (RT-PCR), using degenerate WNV-specific primers, as described in Allison et al. [35].

### Data analyses

We measured WNV presence during the peak transmission months in both the mosquito vectors (July-September) and the avian hosts (July-October) at each site. For mosquitoes, maximum likelihood estimates and 95% CI for the WNV minimum infection rate (MIR) per 1,000 *Culex* mosquitoes were calculated using the Excel [47] Pooled Infection Rate Version 3.0 Add-In [48]. For birds, serological results only from hatch-year individuals were considered, as only they could be reliably confirmed to have been infected during each sampling year [49, 50].

To estimate avian species diversity, we used the R [51] package *vegan* [52] to calculate the Shannon-Wiener diversity index (*H'*) in each site:

$$ {H}^{\mathit{\hbox{'}}}=-{\displaystyle \sum_{i=1}^S}{p}_i\  \ln\ {p}_i $$

Where *p*_*i*_ is the relative abundance of species *i* and *S* is the total number of species present [53]. This measure of diversity was selected as it considers both species richness (number of species) and evenness (abundance) in its calculation.

We tested the relationship between avian species diversity and both avian seroprevalence and mosquito infection rates to see whether a negative relationship existed between the two by modeling the association between avian seroprevalence and multiple predictor variables, including: species diversity, mosquito infection, and microhabitat type using a negative binomial generalized linear mixed model (GLMM) in the R package *glmmADMB* [54], with random effects placed on the site-blocks and years. This model was repeated swapping mosquito infection and avian seroprevalence as dependent and independent variables, respectively.

Finally, given recent evidence [55] that host species diversity experienced by the pathogen (as measured by the host species that *Culex* mosquitoes feed on) may be different from host species diversity at-large (as measured by the host species observed in a point count), we recalculated our avian species diversity measures and data analyses to include only species observed to have been utilized as a host in a previous study examining *Culex* blood-meals, conducted at the same sites and during the same time period as the current study [39].

## Results

During the 2-year study period between July and October of 2010–2011, we took blood samples from 78 wild, unique hatch-year birds, representing 18 species (Additional file 1: Table S1). Overall, 20 (25.6%) birds were seropositive for WNV antibodies, but seroprevalences ranged widely from 0 to 100% between sites and years (Table 1). The highest total seroprevalence was from the Residential microhabitat types while the lowest was from the Forest Patch microhabitat types. Over the same two years between July and September, we collected 26,454 female *Culex* mosquitoes (Table 2) that were aggregated into 1,710 pools and WNV was isolated from 80 (4.7%) pools. Maximum likelihood estimates for the WNV MIR in *Culex* mosquitoes by habitat and year ranged from 1.11 to 7.09 per 1,000. Total MIRs ≥ 5.0 and ≤ 2.5 were recorded from both sites at each microhabitat type, except for the Residential type, where the lowest total MIR was 3.87. Furthermore, the Residential microhabitat type also had the highest total MIR observed at 6.52.

**Table 1:** Hatch-year birds tested for WNV antibodies in four microhabitat types of Atlanta, GA, July-October, 2010–2011

Site name	Habitat type	2010			2011			Total		
		*N*	No. positive	% positive	*N*	No. positive	% positive	*N*	No. positive	% positive
FBB	Forest	8	1	12.5	5	0	0.0	13	1	7.7
WW	Forest	na	na	na	1	0	0.0	1	0	0.0
Total	Forest	8	1	12.5	6	0	0.0	14	1	7.1
GPW	Woods	6	2	33.3	8	2	25.0	14	4	28.6
PPN	Woods	na	na	na	6	1	16.7	6	1	16.7
Total	Woods	6	2	33.3	14	3	21.4	20	5	25.0
GPPO	Water	4	3	75.0	19	3	15.8	23	6	26.1
PPPO	Water	na	na	na	2	0	0.0	2	0	0.0
Total	Water	4	3	75.0	21	3	14.3	25	6	24.0
GPR	Res.	2	2	100.0	6	2	33.3	8	4	50.0
PPR	Res.	na	na	na	11	4	36.4	11	4	36.4
Total	Res.	2	2	100.0	17	6	35.3	19	8	42.1
All Sites		20	8	40.0	58	12	20.7	78	20	25.6

*Abbreviation: na* not available

**Table 2:** *Culex* females tested for WNV in four microhabitat types of Atlanta, GA, USA, July-September, 2010–2011

Site	Habitat type	2010					2011					Total				
		Pools	Pos. pools	*N*	MIR	95% CI	Pools	Pos. pools	*N*	MIR	95% CI	Pools	Pos. pools	*N*	MIR	95% CI
FBB	Forest	29	2	297	7.1	1.3–23.7	41	3	579	5.2	1.4–13.9	70	5	876	5.9	2.2–12.9
WW	Forest	na	na	na	na	na	52	1	902	1.11	0.1–5.4	52	1	902	1.1	0.1–5.4
Total^a^	Forest	29	2	297	7.1	1.3–23.7	93	4	1,481	3.2	0.7–9.7	122	6	1,778	3.5	1.1–9.2
GPW	Woods	484	15	6,780	2.26	1.3–3.6	116	5	2,041	2.5	0.9–5.5	600	20	8,821	2.32	1.4–3.5
PPN	Woods	na	na	na	na	na	101	8	1,659	5.1	2.4–9.6	101	8	1,659	5.1	2.4–9.6
Total^a^	Woods	484	15	6,780	2.3	1.3–3.6	217	13	3,700	3.8	1.7–7.6	701	28	10,480	3.7	1.9–6.6
GPPO	Water	325	10	4,768	2.15	1.1–3.8	171	9	3,044	3.1	1.5–5.6	496	19	7,812	2.5	1.6–3.8
PPPO	Water	na	na	na	na	na	55	4	718	5.8	1.9–13.9	55	4	718	5.8	1.9–13.9
Total^a^	Water	325	10	4,768	2.2	1.1–3.8	226	13	3,762	4.4	1.7–9.7	551	23	8,530	4.1	1.7–8.9
GPR	Res.	189	13	3,041	4.5	2.5–7.5	94	5	1,814	2.8	1.1–6.3	283	18	4,855	3.9	2.4–6.0
PPR	Res.	na	na	na	na	na	53	5	811	6.5	2.4–14.5	53	5	811	6.5	2.4–14.5
Total^a^	Res.	189	13	3,041	4.5	2.5–7.5	147	10	2,625	4.7	1.8–10.4	336	23	5,666	5.2	2.4–10.2
All Sites^a^		1,027	40	14,886	4.0	1.5–9.7	683	40	11,568	4.0	1.5–9.3	1,710	80	26,454	4.1	1.8–8.7

*Abbreviation: na* not available

^a^Total MIRs and 95% CIs are means of each habitat type

**Fig. 2:**
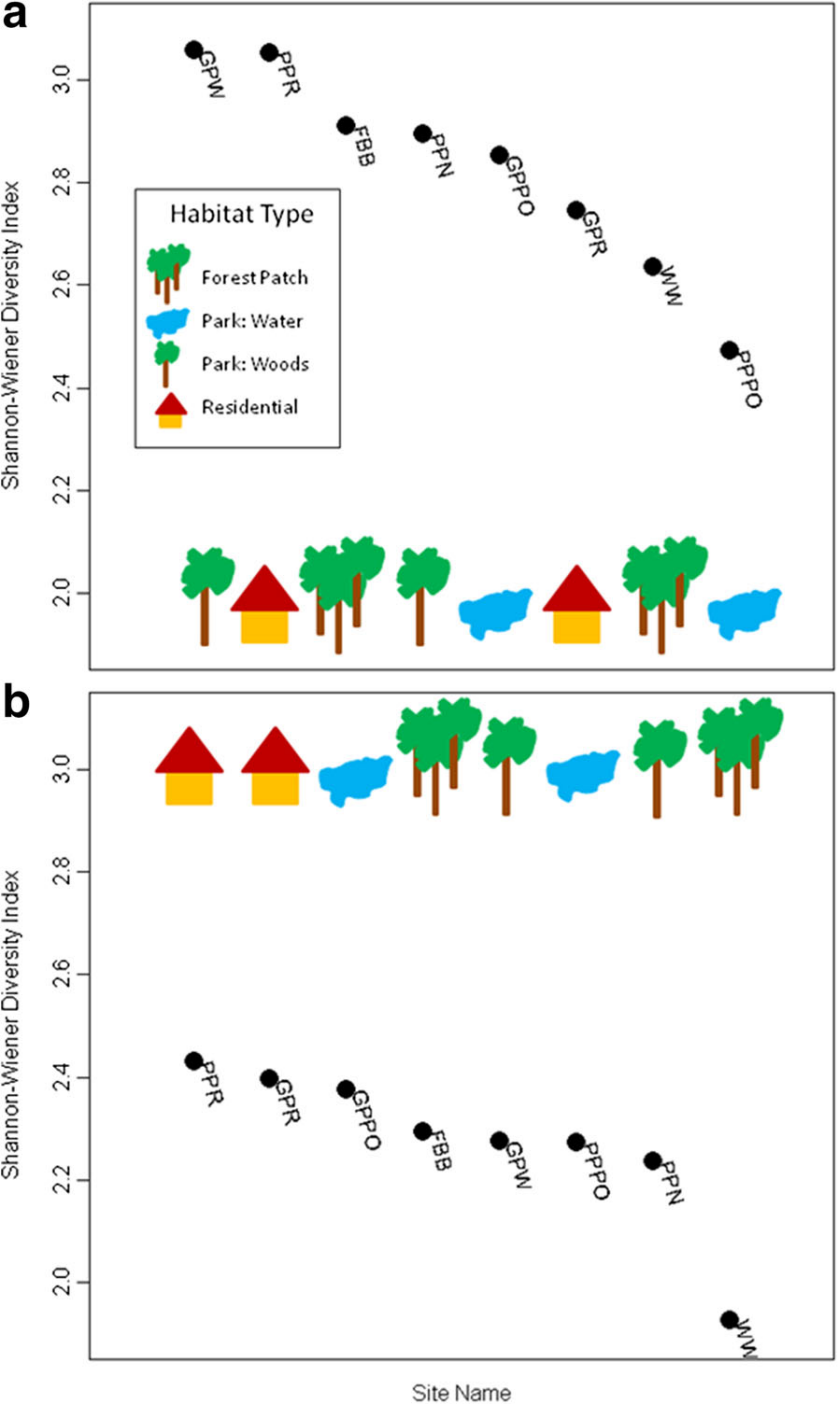
Shannon-Wiener avian species diversity indices. Indices were calculated at each of the eight study sites representing 4 microhabitat types in urban Atlanta, GA, USA, May-October, 2010–2011. **a** Species diversity at-large, calculated considering all observed birds. **b** Species diversity experienced by the pathogen, calculated considering only species observed previously to have been utilized as a *Culex* blood-meal host

**Fig. 3:**
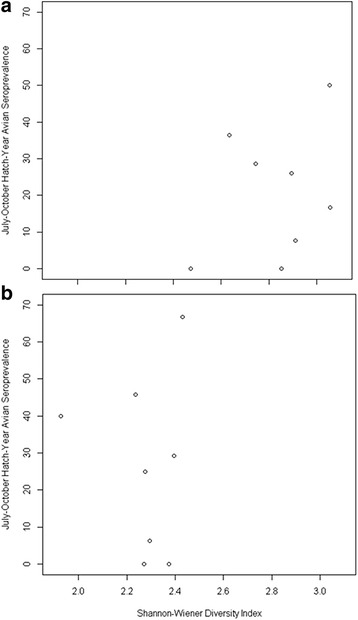
Association between avian species diversity and seroprevalence rates. Included are hatch-year birds at each of the eight study sites representing 4 microhabitat types in urban Atlanta, GA, USA, July-October, 2010–2011. **a** Species diversity at-large: here diversity was calculated considering all observed birds and infection status was examined in all sampled birds. **b** Species diversity experienced by the pathogen: here, both diversity and infection status were calculated considering only species observed previously to have been utilized as a *Culex* blood-meal host

**Fig. 4:**
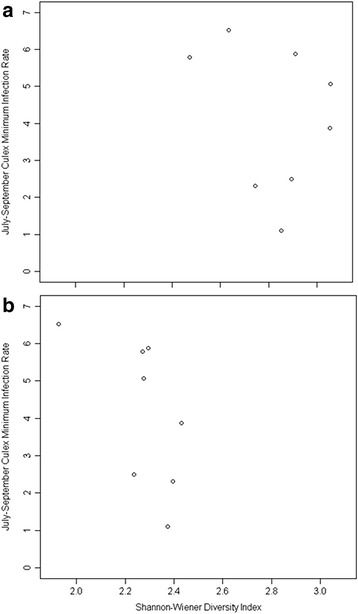
Association between avian species diversity and *Culex* minimum infection rate (MIR). Included are MIRs from each of the eight study sites representing 4 microhabitat types in urban Atlanta, GA, USA, July-September, 2010–2011. **a** Species diversity at-large: here diversity was calculated considering all observed birds. **b** Species diversity experienced by the pathogen: here, diversity was calculated considering only species observed previously to have been utilized as a *Culex* blood-meal host

We conducted 11 point counts at each site over the course of the study and recorded 1,342 birds, representing 64 species. We used these count data to calculate avian species diversity using Shannon-Wiener diversity indices at each site (Fig. 2a). The majority of the most diverse sites were in microhabitat types with the highest tree cover: Forest Patch and Park-Woods, although high diversity was also recorded at one Residential site and low diversity was recorded at one Forest Patch site. We plotted the relationship between avian species diversity and both avian seroprevalence and mosquito infection rates to see whether a negative relationship existed between the two (and hence a possible dilution effect). We observed a slightly positive relationship between diversity and avian seroprevalence (Fig. 3a), and a slightly negative relationship between diversity and mosquito infection (Fig. 4a).

To test this relationship between avian species diversity and avian seroprevalence and vector infection rates, we performed GLMMs to determine the association between infection (of either the host or vector) and multiple predictor variables (species diversity, infection of the other host or vector, and microhabitat type) while controlling for year and site-block. When we considered the model with avian seroprevalence as the outcome variable, we observed a significant (*P* < 0.05) positive association between avian species diversity and avian seroprevalence (Table 3). In addition, there were significantly lower rates of avian seroprevalence from the Forest Patch microhabitats (*P* < 0.01) and significantly higher rates of avian seroprevalence from the Residential microhabitat types (*P* < 0.05). There was no association between mosquito infection rates and avian seroprevalence rates. When we considered the model with mosquito infection as the outcome variable, there were no significant predictor variables (Table 3), although in contrast to the plots, the estimate between mosquito infection and avian species diversity was slightly positive, suggesting that the simple univariate plots fail to capture the true relationship in a complex system.

**Table 3:** Model results for association between diversity of entire sampled avian community and seroprevalence rates. Results from a negative binomial generalized liner mixed model (GLMM) assessing the effects of host seroprevalence or vector infection rate, avian diversity, and microhabitat type on host seroprevalence or vector infection rate from animals captured in urban Atlanta, GA, USA, 2010–2011, while controlling for year and site block. This model considered the diversity of the entire recorded avian community and seroprevalence rates from all sampled avian species

Variable:	Coefficient	Estimate	Std. error	*Z*–value	Pr(>|z|)
	(Intercept)	-5.73	4.56	-1.26	0.21
*Culex* infection	July-September MIR	-0.09	0.13	-0.69	0.49
Avian diversity	Shannon-Wiener Index	3.39	1.68	2.01	0.04*
Habitat^a^	Forest	-2.05	0.70	-2.91	< 0.01**
	Woods	-0.24	0.56	-0.42	0.68
	Res.	1.16	0.50	2.31	0.02*
	(Intercept)	-0.25	2.70	-0.09	0.93
Avian seroprevance	July-October HY seroprevalence	< -0.01	0.01	-0.58	0.56
Avian diversity	Shannon-Wiener Index	0.63	0.99	0.63	0.53
Habitat^a^	Forest	0.03	0.44	0.08	0.94
	Woods	-0.29	0.51	-0.57	0.57
	Res.	0.26	0.47	0.55	0.58

*P < 0.05; **P < 0.01

^a^Coefficient estimates are shown relative to the Water habitat type

To examine the effect of avian species diversity at-large on host seroprevalence and vector infection rates versus the species diversity experienced by the pathogen, we repeated our previous analyses calculating diversity and measuring avian seroprevalence only considering the 24 species (Additional file 1: Table S2) observed previously to have been utilized as a *Culex* blood-meal host from these same sites, during the same period. We recalculated avian species diversity using Shannon-Wiener diversity indices at each site (Fig. 2b). Unsurprisingly, overall diversity dropped at all sites. Additionally, the majority of the most diverse sites shifted from the wooded microhabitat types to the more disturbed sites, with the highest diversity occurring in the Residential and Park-Water sites and the lowest diversity occurring in the Forest Patch and Park-Woods microhabitat types. We again plotted the relationship between diversity and infection rates to see whether a negative relationship existed between the two; however, we observed little relationship between avian diversity and either avian seroprevalence (Fig. 3b) or mosquito infection (Fig. 4b).

To test the relationship between infection rates and avian species diversity as experienced by the pathogen, we again performed GLMMs to determine the association between infection (of either the host or vector) and multiple predictor variables (species diversity, infection of the other host or vector, and microhabitat type) while controlling for year and site-block. When we considered the model with avian seroprevalence as the outcome variable, we again observed a positive relationship between avian species diversity and avian infection (Table 4), although it was not significant (*P* = 0.07). The only significant predictor variable was microhabitat type, in which lower rates of avian seroprevalence were observed from the Forest Patch microhabitat type (*P* < 0.05). There was no association between avian seroprevalence and any other microhabitat types or mosquito infection rates. When we considered the model with mosquito infection as the outcome variable, as with the previous model, we observed no significant predictor variables (Table 4), although the association between mosquito infection and avian species diversity was again positive (*P* = 0.06).

**Table 4:** Model results for association between diversity of utilized sampled avian community and seroprevalence rates. Results from a negative binomial generalized liner mixed model (GLMM) assessing the effects of host seroprevalence or vector infection rate, avian diversity, and microhabitat type on host seroprevalence or vector infection rate from animals captured in urban Atlanta, GA, USA 2010-2011, while controlling for year and site block. This model considered the diversity of the avian community and seroprevalence rates only from avian species also previously identified in at least one *Culex* blood-meal from the same area

Variable:	Coefficient	Estimate	Std. error	*Z*-value	Pr(>|z|)
	(Intercept)	-15.13	10.41	-1.45	0.15
*Culex* infection	July-September MIR	-0.13	0.17	-0.77	0.44
Avian diversity	Shannon-Wiener Index	8.34	4.67	1.79	0.07
Habitat^a^	Forest	-2.19	1.03	-2.14	0.03*
	Woods	1.11	0.69	1.61	0.11
	Res.	0.75	0.59	1.27	0.20
	(Intercept)	-6.11	4.00	-1.53	0.13
Avian seroprevalence	July-October HY seroprevalence	<0.01	<0.01	0.44	0.66
Avian diversity	Shannon-Wiener Index	3.22	1.70	1.89	0.06
Habitat^a^	Forest	0.53	0.47	1.14	0.26
	Woods	0.07	0.45	0.16	0.88
	Res.	-0.09	0.50	-0.18	0.86

*P < 0.05

^a^Coefficient estimates are shown relative to the Water habitat type

## Discussion

In the present study, we aimed to test whether a dilution effect was operating within the WNV host and vector community in various urban microhabitats of Atlanta, GA, USA. Given that the host species diversity experienced by the pathogen (as measured by the host species that *Culex* mosquitoes feed on) may be different from the host species diversity at-large (as measured by the host species observed in a point count), we tested for a negative association between species diversity and infection (in both the hosts and vectors). In a multivariable framework, which controls for ecosystem factors beyond the simple univariate relationship of diversity and infection, regardless of how we measured either avian species diversity or whether we considered host seroprevalence and vector infection predictor variables or outcome variables, we did not detect a negative correlation between species diversity and infection. For the multivariate GLMMs we performed, we observed a consistent positive association between infection and species diversity, which was significant or nearly significant (likely related to our relatively small sample size of hatch-year birds, *n* = 78) in three out of four models. Therefore, we state unequivocally that in the time period and sites sampled in this study, no dilution effect was observed. Rather, we posit that an amplification effect may be operating, in which higher species diversity is associated with increased rates of infection.

Although ours is not the first empirical study to find no evidence of a dilution effect in a fine-scale, urban WNV study, to our knowledge, ours is the first to document what may be an amplification effect occurring in any mosquito-borne pathogen system. While empirical evidence of an amplification effect is rare, the theoretical possibility exists, whereby the presence of multiple hosts may have a multiplicative-type effect on pathogens which makes them more persistent and abundant, even where the hosts are not capable reservoirs [56].

One mechanism for pathogen amplification arising from increased host diversity is the notion that incompetent hosts can increase the abundance of vectors and therefore increase global infection rates. When the hosts in question are either wild or domestic animals that are not the reservoir hosts, this idea is referred to as zoopotentiation [57]. Using simulations of malaria transmission, Saul [57] demonstrated that increasing the number of animal hosts failed to reduce disease transmission when realistic values of vector mortality associated with host-seeking behavior were included in the models. Cohen & Gürtler [58] theoretically showed that an amplification effect would occur in the vector-transmitted Chagas disease system if the triatomine bug vectors had greater numbers of domestic chickens available to feed on, because despite their inability to transmit the pathogen, chickens increased both bug population size and dispersal, ultimately increasing the infected vector population. A similar effect has also been noted in both theoretical models and empirical data from the Lyme disease system, whereby an increased number of the incompetent white-tailed deer hosts can increase disease rates by increasing both the tick vector abundance and infection rate (reviewed by [25]).

Besides contributing to increased vector abundance, a higher diversity of host species, whether competent or not, may also serve to amplify transmission because community composition and/or ecological history rather than absolute host diversity are the key determinants. Randolph and Dobson [25] noted, “Whether dilution or amplification occurs depends more on specific community composition than on biodiversity *per se*.” For example, in a system examining the effect of multiple intermediate hosts on the myxozoan parasites which cause whirling disease in salmonid fish, Steinbach Elwell et al. [59] suggested that rather than the increase in infection they observed by adding another species being due to an amplification effect, it was simply a result of one particular species releasing the other from intraspecific interactions – and that such an effect might not necessarily be observed with a different set of species. Another example of amplification resulting from community composition rather than diversity *per se* was given by Borer et al. [60] where transmission of yellow dwarf viruses among grasses (by their aphid vectors) was increased with the addition of herbivores to the system. In this case, amplification occurred due to an additional guild (consumers) being added to the system rather than because of an increase in the number of species present. Finally, other community ecological factors, such as the timing of pathogen establishment in the presence of other pathogens may determine infection prevalence rates rather than diversity. Using two trematode parasites in the larval stage of an amphibian host, Hoverman et al. [61] showed that the sequence of the addition of the parasites determined their differential infection success as a result of both inter- and intra-specific competition, and that it was the identity of the parasite that mattered more than the number of parasite species.

Following the notion that more hosts (regardless of their competence) can amplify rather than dilute pathogen transmission, we suggest that the possible WNV amplification effect we detected in Atlanta may be due in part to the composition of hosts during the WNV enzootic period. Levine et al. [39] observed that frequent *Culex* mosquito feeding on the moderately competent northern cardinal and three poorly competent species in the Mimid family (northern mockingbirds, brown thrashers, gray catbirds) during August and September dampen WNV transmission in Atlanta. In contrast, other regions receiving larger epizootic transmission events observe more *Culex* feeding on the highly competent American robin [55, 62–64].

One of the four conditions established as necessary for the dilution effect to operate is that optimal hosts are common and widespread [17]. We tested this assumption following Loss et al. by regressing previously modeled abundances [39] of eight common avian species in Atlanta (American robins, blue jays, brown thrashers, Carolina wrens, Cooper’s hawks, house finches, northern cardinals, northern mockingbirds, and song sparrows) on their reservoir competence indices [49, 65]. For this necessary dilution effect condition to be satisfied, we would expect to observe a positive relationship between competence and relative abundance. Instead, we found associations that were not significant between relative abundance and competence in all four microhbaitat types, translating into negligible correlations (*R* ^*2*^ values < 0.08) in all sites but the Park-Woods microhabitat type (*R* ^2^ = 0.39), indicating that optimal hosts are neither common nor widespread in urban Atlanta, and are most likely to occur only in communities with high species diversity. Therefore, higher diversity should amplify rather than dilute transmission [32], a result which is supported by the findings of this study.

An additional possible reason for a failure to observe evidence of a dilution effect in our study may result from the violation of another of its necessary conditions; that vector species must be generalist foragers with no host feeding preference [17]. Previous evidence suggests this condition may be erroneous for *Culex* species, as several studies have demonstrated a marked feeding preference for some avian species over others [63, 66]. *Culex* blood-feeding results from a study utilizing the same sites in Atlanta identified significantly greater feeding from just three out of 41 identified species that provided *Culex* blood-meals: northern cardinals, American robins, and humans. Therefore any correlations between diversity and infection could be spurious, suggesting either dilution (with a highly competent host such as the American robin) or amplification (as we observed with a moderately competent host such as the northern cardinal), when in fact neither would be valid. The final two conditions for the dilution effect of transmission occurring primarily through a vector, and host competencies varying among species, are well established [49].

Finally, as an alternate mechanism of WNV amplification rather than dilution, Roche & Guégan [67, 68] recently theoretically showed that such an effect would be possible in the WNV system with an increase in vector species richness rather than an increase in incompetent hosts. In our study area, based on our previous unpublished data, no evidence suggests that any mosquito species besides *C. quinquefasciatus* and *C. restuans* contribute substantially to WNV transmission. Nevertheless, because *Culex* species in the area belong to a complex, we cannot rule out that additional cryptic species may participate in transmission and therefore contribute to a possible amplification effect through increased vector diversity.

Surprisingly, in addition to observing a possible amplification effect instead of a dilution effect in Atlanta, we also observed no effect of vector infection rate on host seroprevalence rate or *vice versa*. We suspect that this finding is the result of relatively uniform mosquito infection rates across all sites, as shown in a previous study from the area [39]. Furthermore, congruent with those previous findings, significantly reduced avian seroprevalence rates were consistently detected in the Forest Patch habitats. Earlier, we proposed that this result may be due to a higher prevalence of moderately to poorly competent hosts in these habitat types. In light of the findings from this study, similar to results documented in the Chagas disease amplification model, the abundance of poor hosts may decrease local infection rates in the forest patch sites, but increase global infection rates in the greater urban area [58]. These findings indicate the need for further research to explore the scale on which WNV amplification effects occur and whether our results are unique to a region with historically low spillover transmission or a common phenomenon that has not been detected before simply due to the scale of previous studies.

## Conclusions

This study demonstrates for the first time a possible amplification effect rather than a dilution effect for WNV transmission occurring between the host and vector species of urban Atlanta, GA. We provide empirical evidence in support of amplification effects that may primarily be due to WNV transmission in Atlanta being largely driven by abundant moderately to poorly competent host species, such as northern cardinals and mimids, as opposed to highly competent host species such as American robins. When a system is dominated by sub-optimal hosts, optimal hosts become rarer and present mainly in species-rich sites, supporting the notion that dilution or amplification depends more on the identity of hosts rather than the absolute diversity of hosts. We suggest that future studies in Atlanta and elsewhere, which attempt to test the dilution effect, devote particular attention to host species community composition in addition to overall measures of diversity.

## Additional file

Additional file 1: Sampling scheme. **Table S1.** Hatch-year avian species and the number of unique individuals sampled in urban Atlanta, GA, July-October, 2010–2011. **Table S2.** Avian species included in the diversity analysis when considering only species appearing in a previous *Culex* blood-meal from urban Atlanta, GA, May-October, 2010–2011. Stars indicate the 12 species that were also included in the infection analyses (number of individuals sampled are shown in Table 1) from urban Atlanta, GA, July-October, 2010–2011. (DOCX 20 kb)

## Abbreviations

b-ELISA: Epitope-blocked enzyme-linked immunosorbent assay
CPE: Cytopathic effects
GLMM: Generalized linear mixed model
MIR: Minimum infection rate
NIH: National Institutes of Health
RT-PCR: Reverse transcription polymerase chain reaction
SCWDS: Southeastern Cooperative Wildlife Disease Study
WHO: World Health Organization
WNV: West Nile virus
