# Supplementary material for: Avian species diversity and transmission of West Nile virus in Atlanta, Georgia
Source: Parasit Vectors. 2017 Feb 3;10:62. doi: 10.1186/s13071-017-1999-6 (PMC5291963; doi:10.1186/s13071-017-1999-6)
Supplement: Additional file 1: — Sampling scheme. Table S1. Hatch-year avian species and the number of unique individuals sampled in urban Atlanta, GA, July-October, 2010–2011. Table S2. Avian species included in the diversity analysis when considering only species appearing in a previous Culex blood-meal from urban Atlanta, GA, May-October, 2010–2011. Stars indicate the 12 species that were also included in the infection analyses (number of individuals sampled are shown in Table 1) from urban Atlanta, GA, July-October, 2010–2011. (DOCX 20 kb) [file 13071_2017_1999_MOESM1_ESM.docx]

**Additional file 1.** Sampling scheme

During 2010, sampling began in mid-May and continued through the end of October. Each habitat type was represented by a single replicate, and was sampled in the same order. Each site was sampled once every 3 weeks for birds and twice every 3 weeks for mosquitoes. Since mosquitoes were sampled twice as frequently as birds, one of the mosquito trapping sessions at each site occurred on the night prior to avian sampling and one session occurred between avian sampling events. The residential and park sites were represented by the Grant Park (Atlanta’s oldest and fourth-largest urban park) area, which was selected based on its previous determination as a WNV hotspot and the residents’ familiarity with previous WNV surveillance studies (Vazquez Prokopec, Eng et al. 2010). Sampling in the old-growth forest patch was conducted at Fernbank Forest. In the Grant Park residential zone, samples were collected from 10 properties.

In 2011, sampling began in early May and continued through early November. We continued sampling at all the sites from 2010 and added a replicate site for each habitat type. The additional residential and park sites were represented by the Piedmont Park (Atlanta’s third-largest urban park) area, and the additional old-growth forest patch by Wesley Woods. These areas were selected specifically as the best habitat-matches when compared with the 2010 sites. With the addition of the site replicates in 2011, we reduced the frequency of sampling in each site to once every 4.5 weeks for birds and twice every 4.5 weeks for mosquitoes. All sites were again sampled in the same order throughout the season. Samples were collected from 11 properties and 1 community garden in the Piedmont Park residential zone and from 8 properties in the Grant Park residential zone.

**Additional file 1. Table S1.** Hatch-year avian species and the number of unique individuals sampled in urban Atlanta, GA, July-October, 2010-2011.

| **Species Common Name** | **Species Name** | ***N*** |
| --- | --- | --- |
| northern cardinal | *Cardinalis cardinalis* | 29 |
| American robin | *Turdus migratorius* | 17 |
| Carolina wren | *Thryothorus ludovicianus* | 5 |
| European starling | *Sturnus vulgaris* | 7 |
| blue jay | *Cyanocitta cristata* | 3 |
| brown thrasher | *Toxostoma rufum* | 2 |
| eastern towhee | *Pipilo erythrophthalmus* | 2 |
| gray catbird | *Dumetella carolinensis* | 2 |
| northern mockingbird | *Mimus polyglottos* | 2 |
| chestnut-sided warbler | *Setophaga pensylvanica* | 1 |
| downy woodpecker | *Picoides pubescens* | 1 |
| indigo bunting | *Passerina cyanea* | 1 |
| mourning dove | *Zenaida macroura* | 1 |
| rose-breasted grosbeak | *Pheucticus ludovicianus* | 1 |
| song sparrow | *Melospiza melodia* | 1 |
| Swainson’s thrush | *Catharus ustulatus* | 1 |
| white-Throated sparrow | *Zonotrichia albicollis* | 1 |
| wood thrush | *Hylocichla mustelina* | 1 |
| **Total** |  | **78** |

**Additional file 1. Table S2.** Avian species included in the diversity analysis when considering only species appearing in a previous *Culex* blood-meal from urban Atlanta, GA, May-October, 2010-2011. Stars indicate the 12 species that were also included in the infection analyses (number of individuals sampled are shown in table 1) from urban Atlanta, GA, July-October, 2010-2011.

| **Species Common Name** | **Species Name** | |
| --- | --- | --- |
| American robin | *Turdus migratorius* | * |
| blue jay | *Cyanocitta cristata* | * |
| brown thrasher | *Toxostoma rufum* | * |
| brown-headed cowbird | *Molothrus ater* |  |
| Carolina wren | *Thryothorus ludovicianus* | * |
| common grackle | *Quiscalus quiscula* |  |
| Cooper's hawk | *Accipiter cooperii* |  |
| eastern towhee | *Pipilo erythrophthalmus* | * |
| European starling | *Sturnus vulgaris* | * |
| gray catbird | *Dumetella carolinensis* | * |
| house finch | *Haemorhous mexicanus* |  |
| house sparrow | *Passer domesticus* |  |
| house wren | *Troglodytes aedon* |  |
| muscovy duck | *Cairina moschata* |  |
| northern cardinal | *Cardinalis cardinalis* | * |
| northern mockingbird | *Mimus polyglottos* | * |
| pileated woodpecker | *Dryocopus pileatus* |  |
| red-eyed vireo | *Vireo olivaceus* |  |
| rock pigeon | *Columba livia* |  |
| song sparrow | *Melospiza melodia* | * |
| Swainson’s thrush | *Catharus ustulatus* | * |
| tufted titmouse | *Baeolophus bicolor* |  |
| white-breasted nuthatch | *Sitta carolinensis* |  |
| wood thrush | *Hylocichla mustelina* | * |
